# Co-stimulatory signal deficiency impairs cytotoxic T lymphocyte function in tumor immune evasion: molecular mechanisms and therapeutic implications

**DOI:** 10.1186/s12967-026-08711-z

**Published:** 2026-07-28

**Authors:** Junjie Ma, Jing Chen, Tianrun Miao, Yehong Li, Yongmeng Li, Lei Zhao, Hui Tian

**Affiliations:** Shandong Key Laboratory of Digital Diagnosis and Treatment of Thoracic Oncology, Shandong Engineering Research Center of Intelligent Surgery, The First Affiliated Hospital of Shandong First Medical University & Shandong Provincial Qianfoshan Hospital, No. 16766 Jingshi Road, Jinan, 250014 China

**Keywords:** Cytotoxic T lymphocytes (CTLs), Co-stimulatory signals, B7 family, Immune anergy, Tumor immune evasion, Apoptosis, Immune checkpoints

## Abstract

The generation and maintenance of effective tumor-specific CTL responses require more than antigen recognition. In most settings, TCR engagement must be accompanied by co-stimulatory input, with the B7-1/B7-2–CD28 axis being one of the best-characterized examples. When this second signal is weak or absent, tumor-reactive CD8+ T cells may recognize tumor antigens but fail to expand, survive, or acquire and sustain cytotoxic activity. Tumors take advantage of this vulnerability by reducing co-stimulatory ligand availability, increasing inhibitory checkpoint signaling, and remodeling the tumor microenvironment in ways that further restrict T-cell activation. Depending on the stage and context of dysfunction, this shift may favor anergy-like dysfunction, impaired persistence and apoptotic attrition, or, under persistent antigen exposure and sustained inhibitory signaling, exhaustion-associated dysfunction, thereby promoting immune escape. This review examines how reduced or functionally restricted B7–CD28 co-stimulation impairs CTL activation, intratumoral reactivation, and persistence, and how checkpoint signaling, suppressive immune cells, metabolic stress, and stromal barriers compound this defect within tumors. We also evaluate strategies intended to restore or bypass inadequate co-stimulatory input, distinguishing established checkpoint-based interventions from co-stimulatory agonists, engineered T-cell therapies, multispecific antibodies, and gene-based approaches that remain preclinical or early translational in many settings. We propose that mechanism-matched therapy should be guided by the phase at which the dominant barrier arises—tumor-reactive CD8+ T-cell priming, intratumoral CTL reactivation, or long-term CTL persistence.

## Introduction

The generation and maintenance of effective anti-tumor CTL responses depend on coordinated antigen recognition and co-stimulatory signaling. During priming, Signal 1 is delivered when naïve tumor-reactive CD8+ T cells recognize tumor-derived peptides presented on major histocompatibility complex class I molecules by professional antigen-presenting cells through the T-cell receptor (TCR). Signal 2, or co-stimulation, is provided most prominently through the interaction between B7-1/B7-2 (CD80/CD86) on antigen-presenting cells and CD28 on T cells. When this second signal is inadequate, TCR engagement alone is usually insufficient to support productive activation and differentiation of tumor-reactive CD8+ T cells. Instead, tumor-reactive T cells may develop anergy-like dysfunction or reduced survival capacity; under persistent antigen exposure and sustained inhibitory signaling, they may additionally acquire exhaustion-associated dysfunction. Failure of productive co-stimulation is therefore an important mechanism through which tumors weaken immune surveillance [1–4].

The tumor microenvironment (TME) amplifies co-stimulatory deficiency through several overlapping mechanisms. Tumor cells and tumor-infiltrating immune cells may reduce the availability of co-stimulatory ligands, promote suppressive cytokine production, and create conditions that favor T-cell dysfunction. Stromal and immune components within the TME, including tumor-associated macrophages (TAMs) and cancer-associated fibroblasts (CAFs), can further restrain CTL infiltration and activity [5]. In parallel, tumor cells and other TME-resident cells can upregulate PD-L1 and other immunoregulatory B7-family molecules, including B7-H3 and B7-H4, thereby adding inhibitory pressure to local CTLs [6–8]. Deficient co-stimulation therefore commonly arises alongside cytokine-mediated suppression and co-inhibitory signaling rather than as an isolated lesion.

Metabolic changes within the TME add another layer of CTL suppression. Tumor-derived lactate can impair T-cell effector function by disrupting metabolic programs required for proliferation and cytotoxicity [9]. Tumor-intrinsic factors such as NAC1 may reinforce this process by increasing lactate dehydrogenase A (LDHA) expression, promoting lactate accumulation, and strengthening local immunosuppression [10]. These metabolic pressures can further restrict local CTL function when they coexist with deficient co-stimulation and inhibitory checkpoint signaling [9, 10].

Recognition of these mechanisms has encouraged the development of therapeutic approaches that restore, replace, or bypass defective co-stimulatory signaling. Chimeric antigen receptor (CAR) T cells incorporating co-stimulatory domains such as CD28, 4-1BB, or OX40 show improved proliferation, persistence, and cytotoxic activity [11–13]. Other approaches, including bispecific antibodies and nanocarrier-based delivery of immune-activating molecules, are being explored to enhance T-cell activation within the TME [14, 15]. Many of these approaches are also being explored in combination with strategies that target additional suppressive elements of the TME [16–18].

This review examines the role of co-stimulatory signaling in CTL activation, survival, and sustained effector function; the mechanisms by which tumor cells and TME components impair these pathways; and the consequences for anergy-like dysfunction, apoptotic attrition, exhaustion-associated dysfunction, and resistance to immunotherapy. We further evaluate approaches that restore, replace, or bypass inadequate co-stimulatory input.

Our central argument is that the barriers limiting productive anti-tumor CD8+ T-cell responses are phase-specific, and that co-stimulatory failure is a central, but not exclusive, lesion within this continuum. During priming, defective antigen presentation and dendritic-cell maturation can limit the generation of a tumor-reactive CD8+ T-cell pool, whereas restricted B7 availability directly limits co-stimulatory support. During intratumoral reactivation, checkpoint dominance and regulatory-cell-mediated restriction of B7 accessibility may predominate; however, the cellular sources and spatial accessibility of the local co-stimulatory input required to sustain this phase remain insufficiently defined [19, 20]. During prolonged tumor exposure, metabolic stress or inadequate survival signaling may compromise CTL persistence.

## The two-signal model of tumor-reactive CD8+ T-cell activation

### Antigen recognition signal (signal 1)

During priming, recognition of tumor-derived peptides presented as peptide–MHC class I complexes by professional antigen-presenting cells provides Signal 1 to naïve tumor-reactive CD8+ T cells and initiates their differentiation into effector CTLs. Subsequent recognition of peptide–MHC class I complexes on tumor cells enables effector CTLs to exert cytotoxic function. TCR binding triggers the CD3 signaling cassette and initiates downstream signaling pathways that shape T-cell activation, proliferation, and differentiation into effector CTLs [21]. The strength and duration of TCR signaling further shape the quality of this response, influencing effector differentiation and long-term functional persistence.

Critically, Signal 1 alone is rarely sufficient to achieve productive CTL activation. In the absence of adequate co-stimulatory “Signal 2,” TCR engagement may instead lead to an anergic state; exhaustion, by contrast, represents a distinct dysfunction state associated with persistent antigen exposure and sustained inhibitory signaling [3, 4]. Early signaling events following TCR recognition—particularly the formation of signaling microclusters and maturation of the immunological synapse—govern this decision between activation and tolerance. Adaptor proteins such as DLG1 stabilize TCR microclusters and strengthen downstream signal propagation [22], while integrin-mediated “inside-out” signaling, notably through LFA-1, reinforces intercellular adhesion and amplifies TCR-triggered pathways, effectively tuning the activation threshold of T cells [23].

Within the TME, this activation threshold is frequently raised by suboptimal antigen presentation. Tumor cells often express heterogeneous or low-abundance TAAs, limiting the strength of TCR signal transduction. Under these conditions, NK receptors such as DNAM-1 and NKG2D can partially compensate for the absence of CD28 co-stimulation, enhancing TCR signaling sensitivity and effector output, especially when MHC class I expression is reduced [24]. This auxiliary mechanism may explain how CTLs can recognize non-mutated self-antigens and contribute to tumor rejection, and it highlights the therapeutic potential of modulating NK receptor–ligand interactions to restore immunosurveillance.

Signal 1 is also subject to active negative regulation. The immune checkpoint receptor PD-1, upon engaging its ligands, disrupts TCR–CD8 cooperativity and decreases the stability of TCR–pMHC binding, thereby attenuating antigen recognition and T-cell activation [25]. Importantly, PD-1-mediated inhibition preferentially targets CD28 signaling, and the restoration of exhausted CD8+ T-cell responses by PD-1 blockade requires intact CD28/B7 co-stimulation [26, 27]. This inhibitory axis has been co-opted by tumors as a principal means of immune evasion and forms the mechanistic basis for PD-1/PD-L1 blockade therapy. Within tumors, antigen abundance, synapse stability, auxiliary receptor input, and inhibitory checkpoint signaling collectively shape the strength of Signal 1 and the threshold for productive CTL activation [22–27]. Antigen recognition establishes CTL specificity, but effective tumor killing requires that this signal be integrated with adequate co-stimulation and a permissive local immune context [28].

### The necessity of co-stimulatory signals

**Fig. 1:**
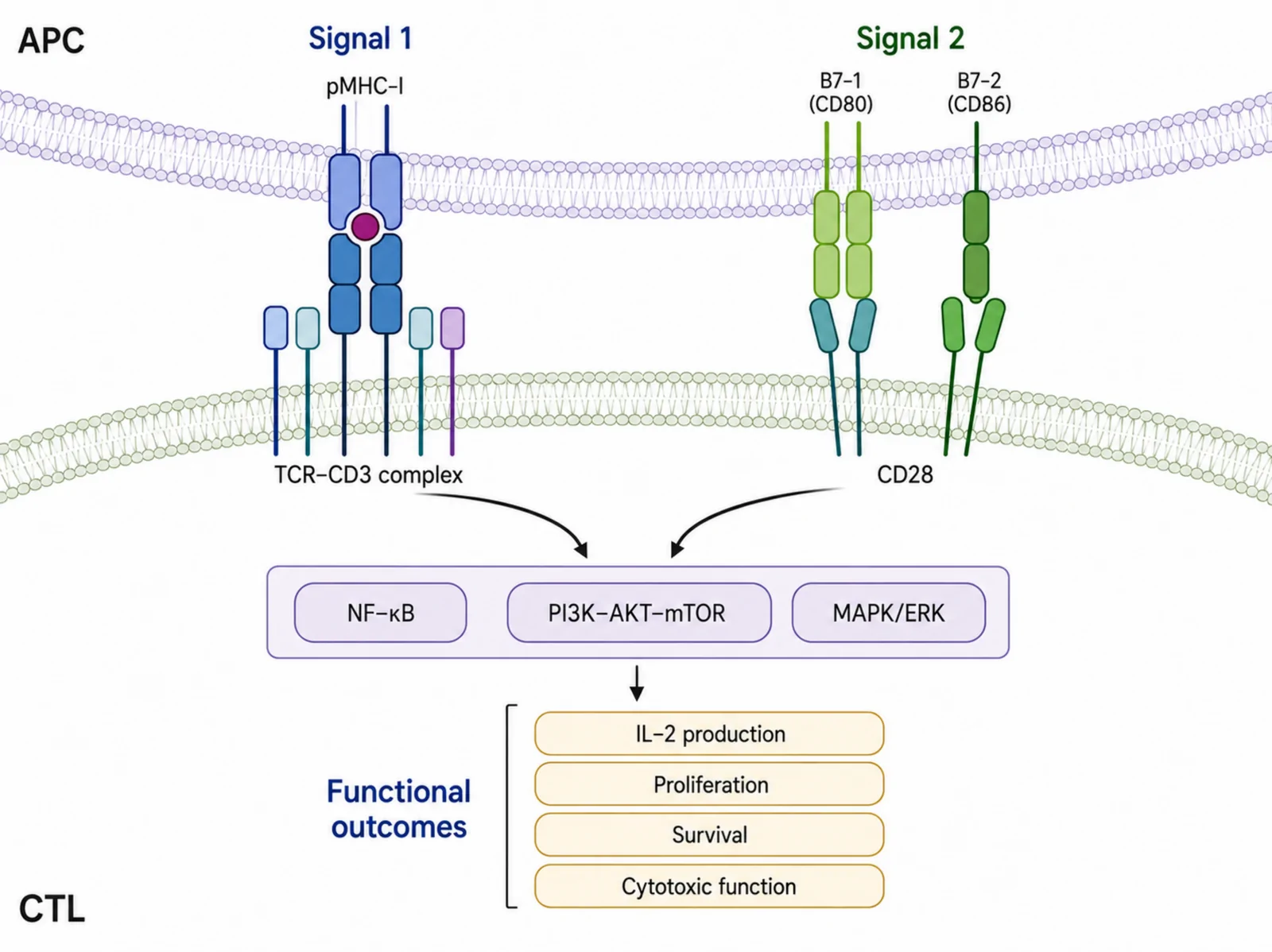
Co-stimulatory signaling in CTL activation. Antigen recognition provides Signal 1 through the interaction between peptide–MHC class I complexes on antigen-presenting cells and the TCR–CD3 complex on CTLs. In parallel, B7-1/CD80 and B7-2/CD86 engage CD28 to deliver Signal 2. Integration of these signals activates NF-κB, PI3K–AKT–mTOR, and MAPK/ERK pathways, supporting IL-2 production, proliferation, survival, and cytotoxic function

Co-stimulatory signaling provides the classical second signal required for optimal T-cell activation, primarily through the interaction of B7-1 (CD80) and B7-2 (CD86) on antigen-presenting cells with CD28 on T cells. Following antigen-specific TCR engagement, CD28 co-stimulation coordinates with TCR-derived signals to promote maximal IL-2 production [2, 29]. IL-2 then supports clonal expansion and effector differentiation, linking CD28 engagement to the magnitude and persistence of the immune response. In the absence of adequate CD28 signaling, TCR engagement alone generally yields limited IL-2 production and proliferation and favors anergy rather than productive activation [2, 3]. As illustrated in Fig. 1, full CTL activation requires the integration of Signal 1, mediated by pMHC–TCR/CD3 recognition, with Signal 2, delivered through B7-1/B7-2–CD28 co-stimulation. The convergence of these signals on NF-κB, PI3K–AKT–mTOR, and MAPK/ERK pathways supports IL-2 production, proliferation, survival, and cytotoxic function.

Beyond its effects on IL-2 production, CD28 co-stimulation can reinforce TCR-proximal signaling. In CAR T cells, CD28-mediated signaling has been shown to increase CD3ζ phosphorylation and ERK activation, thereby increasing the speed and magnitude of activation [30]. Consistently, inadequate CD28 engagement reduces IL-2 production and proliferation even when TCR stimulation is present and can favor anergy [2, 3]. Co-stimulatory dependence varies with T-cell differentiation state and stimulation context. In selected settings, antigen-experienced memory CD8+ T cells can respond to TCR-directed stimulation with reduced dependence on conventional co-stimulation when extrinsic cytokine support is available [31]. This observation should not be generalized to naïve T cells, for which CD28-mediated support remains a major determinant of productive activation [2, 3]. The co-stimulatory requirements of engineered T-cell products depend on receptor design and differentiation state [30–32].

In tumors, APC dysfunction and altered CD80/CD86 expression may reduce the CD28-mediated support available to infiltrating T cells, limiting CTL activation and expansion [33, 34]. CD28-containing CAR-T designs partly bypass this limitation by embedding co-stimulation within the engineered receptor, thereby enhancing activation, expansion, and early effector function after antigen recognition [32]. Bispecific antibodies and fusion proteins that deliver co-stimulatory cues are being developed to augment CTL activity within immunosuppressive TMEs, although the current evidence remains mainly preclinical or early translational [35, 36].

### Co-stimulatory signals and the maintenance of CTL function

Co-stimulatory signaling is not only required for the initiation of T-cell activation but also contributes to the maintenance of cytotoxic T lymphocyte (CTL) function after antigen recognition. Among these signals, CD28 remains the best-characterized pathway. Through its interaction with CD80/CD86 on antigen-presenting cells, CD28 co-stimulation reinforces TCR-triggered signaling and promotes T-cell proliferation [2]. CD28-dependent signaling increases glucose uptake and glycolytic activity in activated T cells, providing metabolic support for proliferation and effector function [37]. These effects are particularly important in tumors, where nutrient restriction, hypoxia, and chronic antigen exposure can progressively weaken CTL activity.

Other co-stimulatory receptors further support CTL persistence and memory formation. Beyond the B7–CD28 axis, TNF receptor superfamily members such as 4-1BB and OX40 can provide additional co-stimulatory input that reinforces CTL expansion, survival, and effector function [38]. 4-1BB stimulation promotes CD8+ T-cell survival by increasing the expression of anti-apoptotic molecules, including Bcl-xL and Bfl-1 [39]. In engineered T-cell settings, OX40-based signaling has also been used to enhance T-cell activity and antitumor responses [13]. 4-1BB signaling can also enhance glucose and fatty-acid metabolism in activated CD8+ T cells, thereby supporting cell-cycle progression and survival [40].

The importance of co-stimulatory signaling is further illustrated by chimeric antigen receptor T-cell design. CAR-T cells incorporating CD28 or 4-1BB co-stimulatory domains show distinct patterns of activation, metabolism, differentiation, and persistence. CD28-containing CARs are generally associated with stronger early activation and glycolytic effector programming, whereas 4-1BB-containing CARs tend to support mitochondrial metabolism, memory-associated differentiation, and longer persistence [41, 42]. These differences have important implications for therapeutic design, particularly in determining whether rapid tumor clearance or long-term T-cell maintenance is the dominant goal. In addition, engineered self-activating co-stimulatory circuits, including CD30- and NF-κB-related signaling modules, have been explored to further enhance CAR-T-cell expansion and anti-tumor activity [43]. These findings emphasize that co-stimulation is closely linked to CTL durability. CD28-, 4-1BB-, and OX40-related signals help connect initial activation with survival, metabolic fitness, and memory potential, which is particularly important in tumors and engineered T-cell therapies where persistence strongly influences therapeutic efficacy.

## Regulatory mechanisms governing co-stimulatory molecule expression in the tumor microenvironment

### Regulation of B7 family molecule expression

Tumors can weaken T-cell activation by altering the expression and functional availability of B7 family molecules within the tumor microenvironment. Among these molecules, B7-1 (CD80) and B7-2 (CD86) are the classical ligands for CD28 and provide the co-stimulatory input required for productive T-cell activation. In tumor tissues, CD80/CD86 expression on APCs can be altered, which may reduce the CD28-dependent input received by local T cells [33, 34]. This loss of co-stimulatory support favors anergy and impaired CTL activation; under persistent antigen exposure and dominant inhibitory signaling, it can also contribute to exhaustion-associated dysfunction [3, 4]. Thus, the regulation of CD80/CD86 expression and accessibility represents a key mechanism through which tumors and tumor-infiltrating immune cells shape the strength of anti-tumor T-cell responses.

However, the B7 family is not limited to classical co-stimulatory ligands. Several non-classical B7 family members, including B7-H3 and B7-H4, are more often discussed as immunoregulatory molecules associated with immune suppression and unfavorable tumor behavior [44, 45]. Unlike CD80/CD86, these molecules do not deliver the classical CD28-mediated co-stimulatory signal; rather, they can reinforce the inhibitory conditions that accompany deficient B7–CD28 co-stimulation. B7-family molecules are also found in tumor-associated macrophages and other myeloid cells, indicating that this regulatory network is not limited to tumor cells or dendritic cells [46, 47].

Metabolic stress within the tumor microenvironment provides another layer of B7 family regulation. Tumor-derived metabolites, particularly lactate, can modulate B7-H3 expression through epigenetic mechanisms such as histone lactylation, thereby weakening CD8+ T-cell activity and promoting immune escape [48]. This link between tumor metabolism and B7 family expression suggests that the metabolic state of the tumor microenvironment is not merely a consequence of tumor growth, but also an active regulator of immune checkpoint and co-stimulatory signaling.

Post-translational regulation further affects the stability, localization, and immunological function of B7 family molecules. For example, palmitoylation of B7-H4 can prevent lysosomal degradation and preserve its immunosuppressive activity [49]. Through these transcriptional, epigenetic, metabolic, and post-translational mechanisms, tumor cells and tumor-infiltrating immune cells can dynamically reshape B7 family signaling in response to immune pressure.

The functional consequences of B7-family regulation depend on the molecule involved. CD80/CD86 availability directly determines the strength of CD28-dependent co-stimulation, whereas B7-H3 and B7-H4 contribute to the suppressive conditions in which CTLs receive that signal through tumor- and myeloid cell-associated mechanisms [44–49].

### Expression and function of tumor-associated B7 family members: B7-H1/PD-L1

B7-H1, more commonly known as programmed death-ligand 1 (PD-L1), is one of the best-characterized immunoregulatory members of the B7 family. PD-L1 is expressed on a wide range of tumor cells as well as on immune and stromal cells within the tumor microenvironment. Upon engaging PD-1 on T cells, PD-L1-mediated inhibitory signaling attenuates TCR–CD8 cooperativity and preferentially suppresses CD28-dependent co-stimulation, thereby limiting CTL proliferation and cytokine production [25–27]. In this way, the PD-1/PD-L1 axis can limit anti-tumor immunity and promote immune escape.

PD-L1 abundance is shaped by tumor-intrinsic genetic or epigenetic programs and by inflammatory signals within the tumor microenvironment. IFN-γ is a major inducer of PD-L1 expression on tumor cells and antigen-presenting cells, mainly through JAK/STAT-related transcriptional programs [50]. In addition, 3′-untranslated region polymorphisms can affect PD-L1 mRNA stability, translational efficiency, and protein abundance [51], whereas glycosylation regulates PD-L1 stability, cell-surface localization, and interaction with PD-1 [52].

Although PD-L1 expression is used as a predictive biomarker for checkpoint blockade in several tumor types, its prognostic and predictive value remains context dependent [53, 54]. PD-L1 should therefore be interpreted alongside antigen-presentation competence, immune-cell infiltration, and the availability of residual B7–CD28 co-stimulatory support, rather than as a stand-alone measure of checkpoint dependence [27, 53]. The broader regulatory and clinical implications of the PD-1/PD-L1 axis are discussed in Sect. 4.1.

### Molecular mechanisms underlying co-stimulatory evasion in the tumor microenvironment

Tumor-associated co-stimulatory failure can result from several mechanisms operating at different stages of the CTL response. Some directly restrict B7–CD28 signaling by reducing ligand availability or altering APC function, whereas others impair antigenicity, antigen presentation, or immune priming upstream of co-stimulation. Together, these defects can prevent productive CTL activation and favor hyporesponsiveness; under persistent antigen exposure and sustained inhibitory signaling, they may also contribute to exhaustion-associated dysfunction.

One direct mechanism is the altered expression of co-stimulatory ligands and co-signaling receptors within tumor tissues. Reduced expression of CD80 and CD86 can limit CD28 engagement and weaken the second signal required for full T-cell activation. In non-small cell lung carcinoma, CD80/CD86 expression has also been linked to the local immune context and patient outcome [34]. Rather than exhibiting a uniform loss of CD80/CD86, tumors may remodel co-signaling networks in a disease- and cell-type-specific manner through tumor-intrinsic transcriptional programs, local inflammatory cues, and immunosuppressive factors within the tumor microenvironment.

Epigenetic regulation provides another mechanism by which tumors impair immune activation. Although not all affected genes encode classical co-stimulatory molecules, epigenetic silencing of immune-sensing and antigen-presentation pathways can indirectly weaken the formation of productive T-cell responses. For example, promoter hypermethylation of cGAS and STING in melanoma cells suppresses the expression of these innate immune mediators, limiting tumor immunogenicity and reducing downstream T-cell activation [55]. Similarly, loss or epigenetic repression of antigen-processing and presentation-related regulators such as NLRC5 can decrease MHC class I expression, thereby reducing antigen visibility to CD8+ T cells and impairing productive T-cell priming [56]. These findings suggest that epigenetic remodeling can impair anti-tumor immunity not only by altering individual co-stimulatory molecules, but also by suppressing the broader antigen-presentation and immune-priming machinery.

Aberrant signaling pathways in tumor cells can also interfere with additional co-stimulatory axes. In nasopharyngeal carcinoma, Epstein–Barr virus-induced ectopic CD137 expression can hijack a negative-feedback pathway that downregulates CD137 ligand on antigen-presenting cells, thereby limiting CD137–CD137L-mediated co-stimulation and facilitating immune evasion [57]. This example shows that tumors may impair co-stimulation not only through CD80/CD86 but also by reducing the functional availability of other inducible co-stimulatory ligands.

Altered APC differentiation and phenotype can further reinforce co-stimulatory failure. In non-small cell lung cancer, tumor cells can modulate human CD1c+ conventional dendritic-cell subsets [33]. Together with altered CD80/CD86 expression in the tumor microenvironment [34], these changes may limit effective local co-stimulatory input and create conditions unfavorable for productive CTL activation. Beyond initial priming, effective intratumoral CTL reactivation may require continued access to functionally meaningful co-stimulatory input [19]. This raises a complementary unresolved question: although CD80 and CD86 are the canonical CD28 ligands and are predominantly supplied by activated antigen-presenting cells [19, 20], it remains unknown whether malignant cells can, in defined tumor contexts, express candidate tumor-cell-associated ligands with co-stimulatory potential that functionally engage infiltrating CD8+ T cells. We therefore propose that intratumoral co-stimulation is a spatially compartmentalized and dynamically regulated resource; its cellular sources, receptor–ligand accessibility, and functional consequences require direct validation, particularly because rescue by PD-1 blockade depends on residual B7–CD28 signaling [26, 27].

**Fig. 2:**
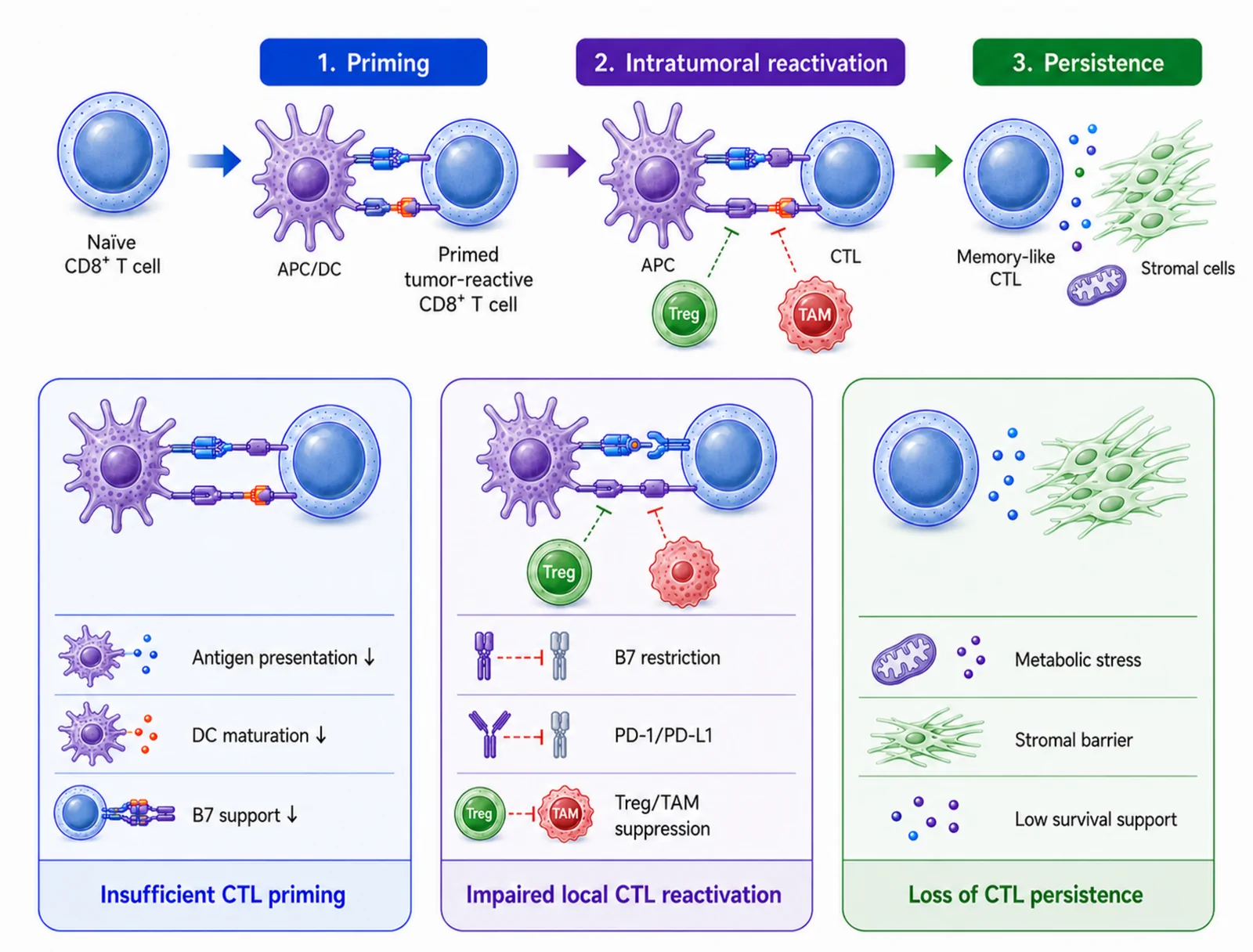
Phase-specific barriers to effective anti-tumor CTL responses. Anti-tumor CD8+ T-cell responses progress from priming through intratumoral CTL reactivation to long-term persistence. During priming, reduced antigen presentation, impaired dendritic-cell maturation, and inadequate B7–CD28 support limit the establishment of an effective tumor-reactive CTL pool. Within tumors, B7 restriction, PD-1/PD-L1 signaling, and Treg/TAM-mediated suppression can impair local CTL reactivation. During the persistence phase, metabolic stress, stromal barriers, and inadequate survival support limit durable CTL function. These barriers may coexist within individual tumors but can predominate at distinct phases of the CTL response

As summarized in Fig. 2, distinct barriers to productive anti-tumor CD8+ T-cell responses can predominate at different phases. Defects in antigen presentation, dendritic-cell maturation, or B7 availability constrain tumor-reactive CD8+ T-cell priming; B7 restriction, inhibitory checkpoint signaling, and Treg/TAM-mediated suppression limit intratumoral reactivation; and metabolic stress, stromal barriers, and inadequate survival support compromise CTL persistence.

Taken together, tumors can impair productive CTL responses through reduced APC-derived co-stimulatory support, epigenetic suppression of antigenicity and immune-priming pathways, and interference with additional co-stimulatory axes [33, 34, 55–57]. These defects should not be treated as therapeutically interchangeable: restoration of CD80/CD86-dependent support may be relevant when B7 accessibility is limiting, whereas impaired antigenicity or antigen presentation requires correction of the upstream priming defect; engineered co-stimulation may be most relevant when endogenous APC support cannot be reliably restored [58].

## CTL anergy and apoptosis in tumor immune evasion

### Definition and induction conditions of T-cell anergy

T-cell anergy is a cell-intrinsic state of antigen-specific functional hyporesponsiveness that develops when TCR engagement occurs under suboptimal conditions, most commonly in the absence of adequate CD28–B7 co-stimulation. Anergic T cells remain viable and retain the capacity to recognize cognate peptide–MHC complexes, yet they fail to mount productive effector responses upon restimulation [3, 59]. The functional signature of this state includes defective interleukin-2 (IL-2) production, failed clonal expansion, attenuated cytotoxicity, and impaired cytokine secretion. Physiologically, anergy serves as a safeguard against autoimmunity by silencing T cells that encounter self-antigens in non-inflammatory contexts. In the tumor microenvironment, however, this same mechanism can be redirected against tumor-reactive cytotoxic T lymphocytes (CTLs). Within the tumor microenvironment, tumor-intrinsic interference with TCR signaling and dominant inhibitory receptor engagement can reinforce this hyporesponsive state [60, 61].

At the biochemical level, anergy reflects an unbalanced TCR signaling state rather than a complete absence of signaling. When Signal 1 is delivered without adequate Signal 2, calcium-dependent NFAT activity can occur without sufficient AP-1 cooperation, favoring an anergy-associated transcriptional program rather than productive IL-2 expression and cell-cycle entry [62]. Thus, anergy does not represent a complete absence of signaling, but rather a partial and unbalanced signal output in which effector-associated transcriptional programs are selectively weakened. This hyporesponsive state can be reinforced by inhibitory receptors. PD-1 engagement raises the activation threshold by attenuating CD28- and TCR-proximal signaling events [25, 26]. When adequate positive co-stimulation is simultaneously lacking, this inhibitory input can reinforce an anergy-like state. However, PD-1 signaling alone should not be equated with classical anergy, which is defined by antigen recognition in the absence of adequate co-stimulatory input [3, 59].

Anergy can also be enforced through direct interference with TCR signal transduction. The HERV-K transmembrane (TM) subunit, a retroviral protein expressed in certain tumors, has been shown to target the CD3ε chain and suppress CD8+ T-cell activation in acute myeloid leukemia and pancreatic ductal adenocarcinoma [60]. This example illustrates that anergy is not exclusively a consequence of missing co-stimulation; it can also be actively imposed by molecular mechanisms that disrupt TCR complex signaling.

The induction and maintenance of anergy in the tumor microenvironment are further promoted by immunosuppressive cell populations. Regulatory T cells (Tregs) can reinforce this state by limiting IL-2 availability and reducing APC co-stimulatory capacity through CTLA-4-mediated removal of CD80/CD86 [63, 64]. Tumor-associated macrophages and tolerogenic dendritic cells can further reduce the availability of co-stimulatory ligands and provide suppressive cytokine cues, thereby shifting local responses away from productive activation [33, 47].

From a therapeutic perspective, restoring productive co-stimulation and relieving dominant inhibitory signaling are complementary strategies for counteracting anergy-like dysfunction. PD-1/PD-L1 blockade can reinvigorate exhausted CD8+ T-cell responses when intact CD28/B7 co-stimulation is available, but it should not be equated with reversal of classical anergy [27].

In tumors, deficient co-stimulation can be compounded by weak TCR signaling, checkpoint engagement, and suppressive immune-cell activity. These interacting mechanisms may generate anergy-like hyporesponsiveness, but they should not be assumed to represent classical anergy in every setting. Restoring CTL responsiveness therefore requires identification of the dominant functional lesion rather than co-stimulatory augmentation alone.

### Apoptotic pathways and their roles in CTLs

Cytotoxic T lymphocytes (CTLs) eliminate target cells through two principal contact-dependent pathways: death receptor engagement and granule exocytosis. The best-characterized death receptor pathway in CTL-mediated killing involves Fas ligand (FasL, CD95L) on the CTL surface binding to Fas (CD95, also known as APO-1) on the target cell. This interaction triggers assembly of the death-inducing signaling complex and activation of the caspase cascade, ultimately leading to apoptotic cell death. In tumor immunity, Fas/FasL signaling has two distinct consequences. CTL-expressed FasL can induce apoptosis in Fas-positive tumor cells, whereas repeated antigen stimulation can render activated tumor-specific CTLs susceptible to Fas/FasL-mediated activation-induced cell death (AICD) [65, 66]. Inadequate CD28 co-stimulation further increases susceptibility to TCR-induced apoptosis and can thereby limit CTL persistence [67]. Within the tumor microenvironment, TGF-β1 can increase PD-1 and CTLA-4 expression on T cells, reinforcing inhibitory signaling and further impairing CTL function [68]. Although these processes may coexist, checkpoint-associated functional suppression should be distinguished from Fas/FasL-mediated apoptotic attrition.

Beyond Fas/FasL, CTLs kill target cells through the directed release of lytic granules containing perforin and granzymes. Perforin facilitates granzyme entry into target cells, while granzyme B activates apoptotic pathways by cleaving Bid, promoting cytochrome c release, and triggering caspase-9 and caspase-3 activation [69]. The biogenesis and polarized trafficking of these lytic granules depend on specialized intracellular machinery. IFT20, a component of the intraflagellar transport system, and TFEB, a transcription factor coordinating lysosomal function, have both been shown to regulate granule biogenesis and secretion; loss of these factors impairs CTL killing capacity [70]. Granzyme A, released alongside granzyme B, contributes to CTL cytotoxicity through a mechanistically distinct route: it cleaves gasdermin B (GSDMB) in target cells to trigger pyroptosis, an inflammatory form of programmed cell death that broadens the consequences of CTL attack beyond apoptosis alone [71].

Tumor cells can evade CTL-induced apoptosis by reducing Fas-dependent death signaling and activating tumor-intrinsic survival pathways [72, 73]. In colorectal cancer, for instance, Fas expression can be silenced through promoter methylation, allowing tumor cells to evade FasL-mediated killing by CTLs [72]. Constitutive activation of STAT3 signaling in tumor cells further contributes to immune escape by suppressing CTL activity and increasing tumor-cell resistance to apoptotic stimuli [73]. These resistance mechanisms do not act in isolation; rather, they operate together with co-stimulatory deficiencies and immunosuppressive networks that already limit CTL fitness within the tumor microenvironment.

**Fig. 3:**
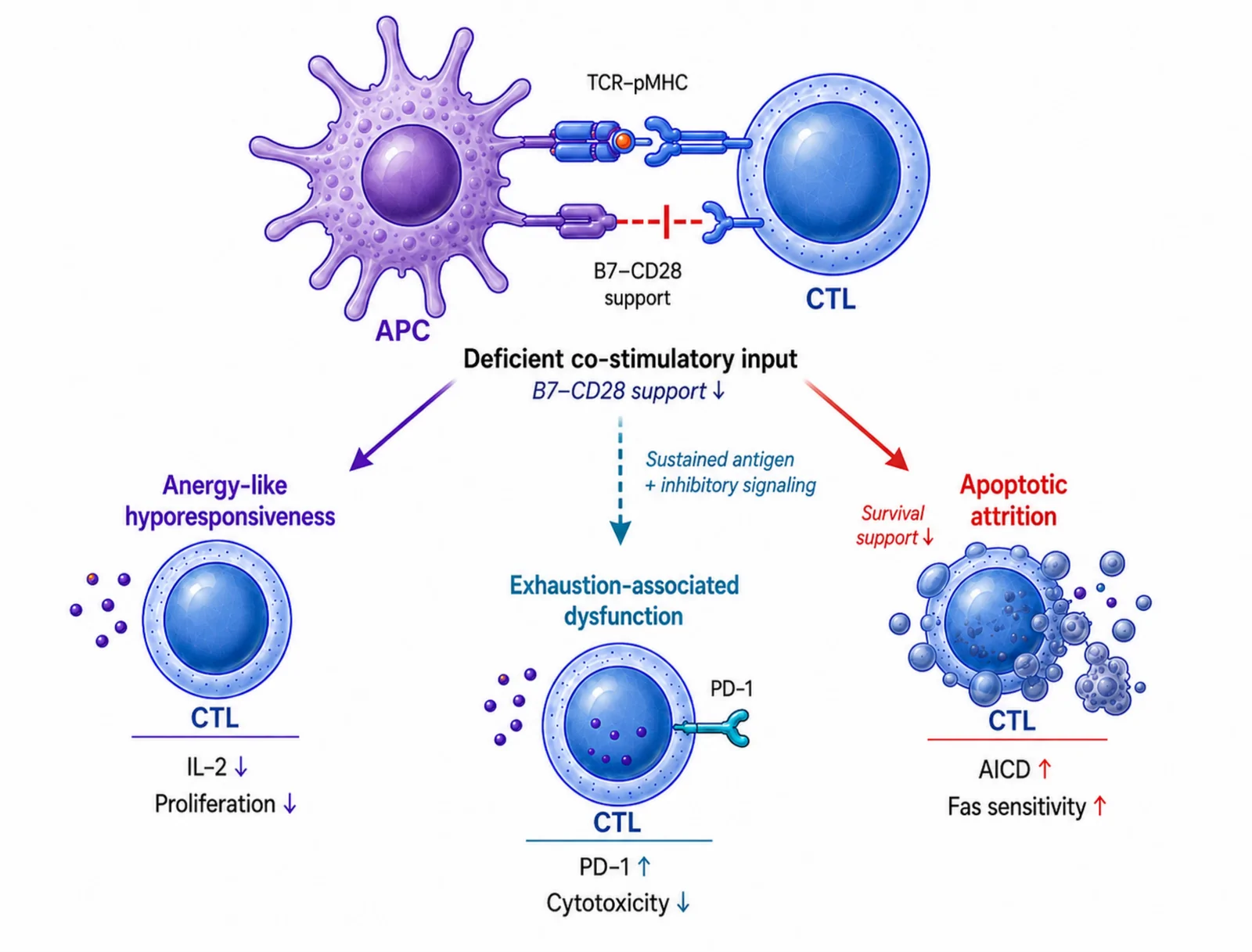
Distinct CTL dysfunctional states associated with deficient co-stimulatory input. TCR–pMHC recognition may remain intact while APC-derived B7–CD28 support is reduced. In this setting, deficient co-stimulatory input can favor anergy-like hyporesponsiveness, characterized by reduced IL-2 production and proliferation, or apoptotic attrition, characterized by reduced survival support, increased activation-induced cell death, and enhanced Fas sensitivity. Under sustained antigen exposure together with persistent inhibitory signaling, co-stimulatory deficiency can also contribute to exhaustion-associated dysfunction, marked by increased PD-1 expression and reduced cytotoxic activity. These dysfunctional states can overlap but are mechanistically distinct

As illustrated in Fig. 3, reduced co-stimulatory input can favor anergy-like hyporesponsiveness [3, 59] and increase susceptibility to apoptotic attrition [67, 74]. Sustained antigen exposure together with persistent inhibitory signaling can promote exhaustion-associated dysfunction [4, 75]. These states may overlap but should not be treated as mechanistically interchangeable.

### Protective anti-apoptotic effects of co-stimulatory signaling

Co-stimulatory signaling, particularly through CD28, is important not only for CTL activation but also for preserving cytotoxic T lymphocyte (CTL) survival during sustained antigen exposure in the tumor microenvironment. CD28 co-stimulation promotes T-cell survival by increasing the expression of the anti-apoptotic protein Bcl-xL [74]. During primary T-cell activation, CD28 co-stimulation can also reduce TCR-induced apoptosis, thereby limiting death-related attrition of activated T cells [67]. By reinforcing pro-survival programs while restraining apoptotic vulnerability, CD28 co-stimulation helps CTLs maintain cytotoxic activity during prolonged immune responses.

Cytokine support can complement, but does not replace, co-stimulatory signaling in the maintenance of CTL survival. IL-12 promotes activated CD8+ T-cell expansion and long-term functional fitness through STAT4-dependent programming [76]. Anti-PD-1-directed low-affinity IL-12, IL-12 prodrugs, and cell surface–tethered IL-12 have enhanced intratumoral CD8+ T-cell activation or adoptive T-cell activity in preclinical studies while aiming to limit systemic exposure [77–79].

The relationship between co-stimulatory signaling, immune checkpoint pathways, and CTL survival is more nuanced than a simple activating-versus-inhibitory model. In an in vitro system of clonally expanding human CD4 + and CD8+ T cells, transient PD-1 engagement during early expansion reduced restimulation-induced cell death (RICD) after strong TCR/CD28 restimulation [80]. This protective effect was weaker in terminally differentiated CD8+ effectors and should not be extrapolated to persistent PD-1 signaling in tumors [80].

By contrast, in tumors, persistent PD-1 engagement is more commonly associated with exhaustion-associated dysfunction, reduced cytokine production, and impaired effector activity [4, 75]. Thus, the effect of co-stimulatory and co-inhibitory signals on CTL survival depends on timing, signal intensity, and the broader immunological context.

These observations help explain why antigen recognition alone is insufficient for durable CTL persistence. When CD28-dependent IL-2 production and pro-survival signaling are inadequate, activated CTLs become more vulnerable to apoptotic attrition; persistent inhibitory signaling can further compromise effector function in tumors [2, 4, 29, 74].

## Roles of immune checkpoint molecules in tumor immune evasion

### The PD-1/PD-L1 pathway

The programmed cell death protein 1/programmed death-ligand 1 (PD-1/PD-L1) pathway is one of the most extensively characterized inhibitory signaling axes in tumor immune evasion. PD-1 is highly expressed on activated and chronically stimulated T cells, including tumor-infiltrating cytotoxic T lymphocytes (CTLs). Upon PD-L1 engagement, PD-1-mediated inhibitory signaling preferentially suppresses CD28-mediated co-stimulation and attenuates TCR–CD8 cooperativity, thereby reducing T-cell activation, proliferation, cytokine secretion, and cytotoxic function [25, 26, 75]. Under persistent antigen exposure, sustained PD-1 signaling contributes to exhaustion-associated dysfunction and undermines CTL-mediated tumor control [4, 75]. By upregulating PD-L1, tumor cells co-opt this pathway to disarm tumor-infiltrating CTLs and establish a locally immunosuppressive microenvironment, positioning the PD-1/PD-L1 axis as a central mechanism of immune escape [53].

PD-L1 abundance is shaped by tumor-intrinsic and microenvironmental signals. Oncogenic pathways can drive constitutive expression, whereas IFN-γ is a major inducible regulator of PD-L1 in the tumor bed. In melanoma models, this response is mediated predominantly through the JAK1/JAK2–STAT1/STAT2/STAT3–IRF1 axis [50]. Stromal crosstalk can further modulate PD-L1 expression; for example, CAF-mediated Akt signaling increases tumor-cell PD-L1 in colorectal cancer [81]. Post-translational regulation, particularly glycosylation, additionally affects PD-L1 stability and immunosuppressive activity [52].

PD-L1-mediated immunosuppression extends well beyond the tumor cell surface. Tumor-associated macrophages (TAMs) suppress perforin and granzyme B expression in CD8+ T cells through PD-1/PD-L1 engagement, weakening CTL cytotoxicity and promoting immune escape in multiple myeloma [82]. During tumor-antigen presentation, dendritic cells can upregulate PD-L1 and limit T-cell priming, placing PD-1-mediated inhibition at the same APC–T-cell interface where B7–CD28 co-stimulation is delivered [83]. Tumor-derived exosomes carrying PD-L1 further extend the reach of this inhibitory signal beyond the local tumor bed, promoting systemic immunosuppression and reducing responsiveness to checkpoint blockade [84, 85]. Together, PD-L1 expression across tumor cells, myeloid cells, DCs, and exosomal compartments creates a redundant and spatially distributed immunosuppressive network.

Therapeutic blockade of PD-1 or PD-L1 represents one of the most significant advances in cancer immunotherapy. By interrupting this inhibitory axis, anti-PD-1/PD-L1 antibodies can partially reinvigorate exhausted T cells, restore cytokine production and cytotoxic activity, and generate durable clinical responses across a range of tumor types [53, 75]. However, not all patients benefit, and both primary and acquired resistance remain major clinical challenges. Resistance can arise from heterogeneous PD-L1 expression, insufficient antigen presentation, exclusion of effector T cells from the tumor parenchyma, compensatory upregulation of alternative checkpoint pathways, immunosuppressive myeloid populations, and metabolic constraints on T-cell function within the tumor microenvironment [86, 87]. These limitations indicate that PD-1/PD-L1 blockade is most likely to benefit tumors in which antigen-specific CTLs are present and residual priming and co-stimulatory capacity are preserved [27]. By contrast, immune-excluded or immune-desert tumors and tumors with defective antigen presentation may require combination strategies that address these additional barriers [86]. Impaired APC differentiation or reduced CD80/CD86 availability may represent an additional co-stimulatory barrier to effective CTL activation [33, 34].

The PD-1/PD-L1 pathway should therefore be considered a distributed suppressive axis rather than a tumor-cell-restricted checkpoint. PD-L1 expression on tumor cells, myeloid cells, dendritic cells, and exosomes can all contribute to CTL inhibition, which helps explain why PD-1/PD-L1 blockade may be insufficient when poor antigen presentation, weak co-stimulation, or parallel suppressive pathways dominate [75, 82–84].

### CTLA-4 and other checkpoint molecules

CTLA-4, also known as cytotoxic T-lymphocyte antigen-4, is an immune checkpoint receptor expressed on activated conventional T cells and constitutively on regulatory T cells (Tregs). It is particularly important at the antigen-presenting cell (APC)–T-cell interface during early T-cell priming, where it limits CD28-mediated co-stimulation. By sharing CD80 and CD86 with CD28, CTLA-4 competitively restricts B7 availability and blocks CD28-dependent activation [88]. Beyond ligand competition, CTLA-4 can capture and remove CD80/CD86 from APCs through trans-endocytosis, further diminishing their capacity to deliver co-stimulation [64]. Through these mechanisms, CTLA-4 can weaken tumor-reactive CD8+ T-cell priming and promote immune escape within tumors.

The immunosuppressive role of CTLA-4 is especially relevant in tumor-associated Tregs. Treg-expressed CTLA-4 can reduce CD80/CD86 availability on APCs through ligand capture and removal, thereby limiting the B7–CD28 co-stimulatory capacity available to tumor-reactive effector T cells [64, 89]. High intratumoral Treg abundance and CTLA-4hi Treg phenotypes have been associated with tumor progression and may influence responsiveness to immunotherapy [90–93]. CTLA-4 blockade may enhance anti-tumor immunity by preserving B7 availability for CD28-mediated activation and, for some antibody formats, by reducing intratumoral Treg-mediated suppression. In preclinical melanoma models, the latter component depended on Fc-mediated depletion of tumor-infiltrating Tregs [91]. However, because CTLA-4 is critical for maintaining peripheral tolerance, its inhibition can provoke immune-related adverse events, including hypophysitis and cutaneous toxicity [94, 95]. These toxicities highlight the need to balance enhanced anti-tumor immunity with preservation of peripheral tolerance.

Beyond CTLA-4, other checkpoint receptors can add parallel inhibitory pressure to tumor-reactive T cells. TIM-3, LAG-3, and TIGIT are associated with impaired T-cell activation, cytokine production, and cytotoxic function in tumor settings [96–100]. Unlike CTLA-4, however, these receptors should not be interpreted as direct mediators of B7–CD28 co-stimulatory ligand loss. Rather, they can intensify CTL dysfunction when productive co-stimulation is already inadequate. Their therapeutic targeting is therefore best viewed as relief of parallel inhibitory pathways rather than direct restoration of B7 availability.

Combined CTLA-4 and PD-1 blockade is clinically validated in selected settings, including advanced melanoma and non-small cell lung cancer, but enhanced anti-tumor activity is accompanied by a higher risk of immune-related toxicity than monotherapy [101–103]. Combinations involving additional checkpoint targets, including LAG-3, remain investigational in most tumor contexts and should be interpreted according to tumor immune state and level of clinical evidence.

CTLA-4 is distinguished from other checkpoint receptors by its direct control of B7 availability at the APC–T-cell interface. This feature is particularly relevant to the phase-specific interpretation of checkpoint blockade, because CTLA-4 can restrict the co-stimulatory substrate required for productive T-cell activation [64, 88].

### Mechanisms and clinical application of immune checkpoint blockade therapy

Immune checkpoint blockade (ICB) interrupts inhibitory receptor–ligand interactions, but the mechanisms it engages are context- and phase-dependent. Experimental evidence indicates that recovery of dysfunctional CD8+ T-cell responses after PD-1 blockade depends on residual B7–CD28 co-stimulation [26, 27]. CTLA-4 blockade can preserve B7 availability at the APC–T-cell interface, particularly during priming, by limiting CTLA-4-mediated competition for and removal of CD80/CD86 [64, 88]. Thus, ICB can relieve inhibitory constraints, but it does not itself restore absent antigen presentation, defective APC function, or loss of local co-stimulatory ligands.

Clinical benefit from ICB is not uniform across tumor types. In classical Hodgkin lymphoma, PD-1 blockade produces high response rates in relapsed or refractory disease [104]. Frequent 9p24.1 copy-number alterations increase PD-L1/PD-L2 expression in this disease, supporting its classification as a PD-1-ligand-dominant context [105]. Durable benefit is also established in subsets of patients with melanoma, non-small cell lung cancer, and renal cell carcinoma [101, 106, 107]. In mismatch repair-deficient or microsatellite instability-high solid tumors, response to PD-1 blockade represents a distinct biomarker-defined clinical context [108]. These tumor settings should not be regarded as a single “ICI-sensitive” category, because they differ in antigenicity, baseline T-cell infiltration, dominant inhibitory mechanisms, and the relative contributions of defective priming and intratumoral effector dysfunction [109].

A practical mechanism-based framework is to distinguish checkpoint-dominant, T-cell-inflamed tumors from priming-defective or immune-excluded tumors. In checkpoint-dominant, T-cell-inflamed tumors, pre-existing tumor-reactive T cells and residual APC-derived co-stimulatory support provide the biological substrate on which PD-1/PD-L1 blockade can act [27, 109]. By contrast, tumors with defective antigen presentation, inadequate CD80/CD86 availability, or abnormal dendritic-cell differentiation may lack the cellular and co-stimulatory substrate required for effective ICB monotherapy [33, 34, 55, 56]. Stromal exclusion and metabolic suppression can further limit therapeutic response [109–111]. These tumors may require combinations that restore antigenicity, productive priming, T-cell trafficking, or local co-stimulatory input rather than additional checkpoint blockade alone. A major clinical limitation of ICB is the occurrence of immune-related adverse events, which commonly involve the skin, gastrointestinal tract, endocrine glands, and liver and vary according to checkpoint target and treatment regimen [103].

Combined PD-1 and CTLA-4 blockade provides a clinically validated example of phase-complementary immunotherapy. In CheckMate 227, first-line nivolumab plus ipilimumab improved overall survival compared with chemotherapy in metastatic non-small cell lung cancer [102]. In advanced melanoma, nivolumab plus ipilimumab has also produced durable long-term survival in a proportion of patients [101]. However, this enhanced activity is accompanied by a greater risk of immune-related toxicity than monotherapy [103]. Other combinations involving chemotherapy, anti-angiogenic therapy, radiotherapy, or photodynamic approaches aim to improve antigen release, T-cell infiltration, or suppressive microenvironmental barriers [110, 112–114]. The evidence supporting these strategies ranges from clinically established regimens in selected diseases to early clinical investigation and preclinical models; therefore, they should not be interpreted as equivalently validated therapeutic approaches.

LAG-3 should not be considered uniformly investigational, because relatlimab plus nivolumab has phase 2–3 clinical evidence in previously untreated unresectable or metastatic melanoma [115]. By contrast, TIM-3-, TIGIT-, and VISTA-directed strategies remain investigational in most tumor contexts, and their clinical utility should be interpreted according to tumor type, biomarker context, and level of clinical evidence [116]. Metabolic features of the TME, particularly lactate accumulation, can impair T-cell function and limit the effectiveness of checkpoint blockade [9, 111]. The composition of the gut microbiota has also been shown to modulate systemic immune tone and ICB responsiveness [117]. Interventions targeting these factors may help convert poorly responsive tumors into more immune-permissive states and support personalized combination strategies.

ICB has established the clinical value of relieving inhibitory constraints on CTLs, but it also exposes a central limitation: checkpoint release does not automatically restore the activating and survival programs required for durable CTL function. Tumors dominated by defective priming, restricted B7 availability, immune exclusion, metabolic stress, or impaired CTL persistence may therefore require mechanism-matched combinations that address the phase at which the CTL response is failing.

## Effects of immunosuppressive factors in the tumor microenvironment

### Regulatory T cells

Regulatory T cells (Tregs) can directly restrict CTL activation at the antigen-presenting cell (APC)–T-cell interface. Through constitutive CTLA-4 expression, Tregs capture and remove CD80/CD86 from APCs, thereby reducing the B7–CD28 co-stimulatory input available to tumor-reactive T cells [64, 89]. Tregs can also limit local IL-2 availability, thereby constraining CTL expansion, and produce IL-10 and TGF-β, which can impair dendritic-cell maturation and reinforce a suppressive local environment [63]. Thus, Treg-mediated suppression is not limited to direct inhibition of effector CTLs; it can impair the local priming and reactivation conditions required for productive CTL responses.

### Tumor-associated macrophages

Tumor-associated macrophages (TAMs) can compromise CTL function through direct checkpoint signaling and indirect impairment of local antigen-presenting cell support. PD-L1 expressed on TAMs can suppress intratumoral CD8+ T-cell effector function through PD-1 engagement [82, 118]. TAM-derived IL-10 can additionally suppress IL-12 expression in intratumoral dendritic cells, thereby weakening the local APC-derived signals required to sustain effective CD8+ T-cell responses [119]. These mechanisms position TAMs at the interface between checkpoint dominance and defective local T-cell reactivation rather than portraying them solely as sources of general inflammation or angiogenic factors.

Because TAM-mediated suppression is heterogeneous across tumors, macrophage-directed interventions should be interpreted according to the dominant suppressive mechanism in a given tumor context. In preclinical pancreatic cancer models, CSF1/CSF1R blockade reprogrammed tumor-infiltrating macrophages, enhanced antigen presentation, and improved responses to T-cell checkpoint immunotherapy [120]. These preclinical findings support macrophage reprogramming as a potential complement to checkpoint blockade, but they do not establish this approach as a clinically validated therapeutic strategy.

### Coordinated suppressive networks of Tregs and TAMs

Tregs and TAMs can impose complementary constraints on CTL activation within the same tumor microenvironment. Tregs directly restrict B7–CD28 co-stimulation through CTLA-4-dependent removal of CD80/CD86 from APCs, whereas TAMs impose PD-L1-mediated inhibition and can diminish intratumoral dendritic-cell support through cytokine-mediated mechanisms [64, 89, 118, 119]. Co-stimulatory failure may therefore reflect both reduced APC ligand availability and the accumulation of parallel myeloid-derived inhibitory signals. This distinction provides a rationale for tumor-context-specific strategies that target Tregs, reprogram TAMs, or address both compartments.

## Co-stimulatory signal deficiency in immunotherapy resistance and therapeutic strategies

### Co-stimulatory signal deficiency and resistance to immunotherapy

The efficacy of immunotherapy depends on the integrity of several sequential steps in the CTL response, including tumor-antigen recognition, antigen-presenting cell (APC)-dependent priming or local reactivation, and the maintenance of effector function within the tumor microenvironment. Co-stimulatory deficiency is most directly relevant to the latter two stages. When B7–CD28 input is inadequate, antigen recognition alone may fail to sustain IL-2 production, clonal expansion, and pro-survival signaling, favoring anergy-like dysfunction and apoptotic attrition [2, 3, 29, 74]. Under persistent antigen exposure and dominant inhibitory signaling, these defects can also contribute to exhaustion-associated dysfunction [4].

Loss of tumor MHC class I antigen presentation lies upstream of co-stimulatory signaling and represents a distinct mechanism of primary or acquired resistance to CD8+ T-cell-based immunotherapy [121]. In melanoma, acquired resistance to PD-1 blockade has been associated with defects in interferon-receptor signaling and antigen-presentation pathways, including loss of β2-microglobulin [122]. However, preserved MHC class I expression does not guarantee effective immunotherapy. Tumor-driven alterations in APC differentiation and local CD80/CD86 availability may still limit productive T-cell priming or intratumoral reactivation [33, 34]. Antigen-presentation failure and co-stimulatory failure should therefore be considered complementary, rather than interchangeable, barriers to CTL-mediated tumor control.

Stromal barriers do not directly cause co-stimulatory failure, but they can compound its functional consequences by limiting the access of activated T cells to tumor regions where antigen recognition and local immune support are required. As an illustrative preclinical example, CD80-Fc fusion protein combined with DDR1 inhibition enhanced T-cell infiltration and anti-tumor activity in gastric cancer models [123]. This finding supports the concept that restoration of co-stimulatory input may need to be combined with stromal remodeling in immune-excluded tumors; however, CD80-Fc/DDR1 inhibition should not be interpreted as a clinically validated therapeutic strategy.

Some approaches may bypass, rather than restore, the classical antigen-recognition and co-stimulatory axis. In preclinical models of MHC class I-negative tumors, cIAP1/2 antagonism induced a T cell–macrophage axis that promoted phagocytic tumor clearance [124]. This mechanism is relevant to tumors with irreversible antigen-presentation defects, but it does not restore B7–CD28 co-stimulation and should be regarded as a preclinical alternative-effector strategy.

Co-stimulatory deficiency should therefore be viewed as one functional bottleneck in immunotherapy resistance. Durable benefit from checkpoint blockade is unlikely when antigen visibility, APC co-stimulatory capacity, stromal access, and CTL survival are concurrently impaired. Mechanism-based combinations should be selected according to the dominant defect—restoring antigenicity, improving APC co-stimulation, overcoming immune exclusion, or supporting CTL persistence—rather than by empirically intensifying checkpoint blockade. A tumor-context-specific framework linking dominant co-stimulatory defects, candidate biomarkers, and therapeutic implications is summarized in Table 1.

**Table 1:** Tumor-context-specific barriers to productive anti-tumor CD8+ T-cell responses, candidate biomarkers, and therapeutic implications

Tumor immune context and representative examples	Dominant barrier to productive anti-tumor CD8+ T-cell responses and affected immune phase	Candidate mechanistically informed biomarkers	Expected implication for immune checkpoint blockade	Mechanism-guided strategy and evidence level
Checkpoint-dominant, inflamed tumors; representative settings include subsets of melanoma, NSCLC, and RCC	Tumor-reactive CTLs, antigen presentation, and residual APC-derived B7–CD28 support are retained, but PD-1/PD-L1 signaling suppresses CD28-dependent effector-phase reactivation	Intratumoral CD8+ T-cell density; PD-L1 distribution; mature DC or APC abundance and spatial proximity to intratumoral CD8+ T cells; CD80/CD86 expression; CD28-positive or progenitor-exhausted CTL subsets	Checkpoint release is more likely to be effective when a recoverable tumor-reactive CTL pool and residual APC-derived co-stimulation are present	PD-1/PD-L1 blockade; selected use of PD-1/PD-L1 plus CTLA-4 blockade; consideration of local co-stimulatory reinforcement when persistence is incomplete Evidence: Clinical benefit from checkpoint blockade is established in selected indications; the APC/B7/CD28 interpretation remains mechanistic and translational [26, 27, 101, 102, 107].
PD-1-ligand-dominant tumors; classical Hodgkin lymphoma as a representative context	Strong PD-1/PD-L1/PD-L2-mediated restraint of pre-existing anti-tumor T-cell responses; co-stimulatory failure has not been established as the dominant barrier in this context.	PD-L1/PD-L2 expression; 9p24.1 copy-number alterations; T-cell infiltration; APC and CTL functional state	PD-1 blockade can generate substantial clinical activity when PD-1-ligand-driven inhibition is a dominant and targetable barrier	PD-1-directed therapy; rational combinations only when additional priming, regulatory, or persistence defects are evident Evidence: Clinical efficacy is established; the contribution of co-stimulatory competence should be interpreted as a contextual modifier rather than a validated clinical biomarker [104,105].
Antigenicity-high tumors, including MSI-H/dMMR tumors; MSI-H/dMMR colorectal cancer as a representative setting	High neoantigen burden and pre-existing immune recognition may establish an inflamed state, but inhibitory signaling can restrain effective CTL reactivation	MSI-H/dMMR status; tumor mutational burden; antigen-presentation competence; immune infiltration; PD-L1 and IFN-γ-related signatures	Checkpoint blockade is more likely to be effective than in poorly primed immune-cold tumors, although not all MSI-H/dMMR tumors respond	PD-1 blockade; assessment of additional resistance mechanisms including antigen-presentation loss, myeloid suppression, and stromal exclusion Evidence: Clinical efficacy of PD-1 blockade is established in selected MSI-H/dMMR solid tumors, including colorectal cancer; co-stimulatory defect classification remains hypothesis-generating [108].
Treg-rich or B7-restricted tumor microenvironment; a cross-histology context rather than a fixed tumor type	CTLA-4-mediated competition, trans-endocytosis, and trogocytosis reduce APC-derived CD80/CD86 availability during priming and local CTL reactivation	Intratumoral Treg density; CTLA-4 expression; CD80/CD86 expression on APCs; cDC1 or mature DC abundance; APC-associated PD-L1 expression.	Checkpoint release alone may be insufficient if B7 accessibility and APC competence remain severely impaired	CTLA-4 blockade; Treg-directed approaches; restoration of APC competence; DC activation or vaccination; local restoration of B7 accessibility Evidence: CTLA-4 blockade is clinically established in selected cancers; B7/Treg-guided selection strategies remain translational or investigational [64, 89, 91, 101, 102].
Immune-cold or priming-defective tumors	Defective tumor antigenicity, MHC class I presentation, DC recruitment, DC maturation, or APC-derived B7–CD28 priming prevents establishment of a competent tumor-reactive CTL pool	MHC class I, B2M, NLRC5, cGAS–STING, or type I interferon-associated signatures; cDC1 or mature DC abundance; T-cell infiltration; chemokine signatures	Limited activity is expected from checkpoint blockade alone because there may be insufficient tumor-reactive CTLs or inadequate APC–B7–CD28 priming substrate	Restoration of antigen presentation when reversible; innate immune activation; DC recruitment or licensing; cancer vaccination; and, for irreversible MHC class I loss, alternative effector strategies rather than checkpoint blockade alone. Evidence: Predominantly preclinical or early clinical; optimal intervention is highly tumor- and platform-dependent [55, 56, 121, 122].
Stromal-, metabolic-, or myeloid-constrained tumors	Local CTL reactivation and persistence are limited by suppressive TAM/CAF programs, hypoxia, lactate accumulation, nutrient restriction, vascular exclusion, and reduced local survival support	TAM and CAF signatures; hypoxia/lactate signatures; vascular exclusion; suppressive cytokine programs; CTL persistence or metabolic stress markers	Responses may be transient or incomplete despite partial checkpoint release because CTLs remain poorly supported within the tumor site	Myeloid or stromal reprogramming; vascular normalization; metabolic remodeling; checkpoint blockade combined with persistence-supporting strategies Evidence: Evidence is mixed; some disease-specific combinations are clinically established, whereas co-stimulation-focused restoration strategies remain investigational [110, 111, 119, 120].
Persistence-limited or adoptive-cell-therapy context	Tumor-microenvironmental support is weak or insufficient to sustain transferred T-cell survival, metabolic fitness, trafficking, and durable function after activation.	CTL apoptosis or persistence signatures; metabolic stress; antigen heterogeneity; trafficking defects; CAR/TCR-T expansion and persistence markers	Checkpoint blockade alone is unlikely to fully compensate for limited transferred T-cell persistence, impaired trafficking, antigen heterogeneity, or poor tumor access.	Engineered CD28- or 4-1BB-based T cells; TCR-T; switch receptors; tumor-localized co-stimulation; locoregional delivery; metabolic and microenvironmental support Evidence: CAR-T is clinically established mainly in hematologic malignancies; solid-tumor and engineered persistence strategies remain early clinical or preclinical [41, 42, 130, 131].

Abbreviations: APC, antigen-presenting cell; CAF, cancer-associated fibroblast; cDC1, type 1 conventional dendritic cell; CTL, cytotoxic T lymphocyte; dMMR, deficient mismatch repair; MSI-H, microsatellite instability-high; RCC, renal cell carcinoma; TAM, tumor-associated macrophage; B2M, beta-2 microglobulin

These categories are analytical tumor-immune contexts rather than fixed histological classifications and are not mutually exclusive. Candidate biomarkers are mechanistically informed variables and should not be interpreted as clinically validated standalone selection criteria. Mechanistic interpretations and evidence levels are based on the studies discussed in Sect.  2.3, 4.2–4.3, and 5.1–6.3. Current evidence is also insufficient to quantify the relative prevalence of priming-, intratumoral reactivation-, and persistence-phase defects across tumor types. Accordingly, the phase assignment in this table reflects the biologically predominant site of action of each barrier rather than a numerical frequency estimate

### Combination immunotherapy strategies

Combination immunotherapy should be selected according to the dominant barrier to CTL function rather than used as empirical treatment escalation. In tumors in which antigen-specific CTLs are present but remain constrained by inhibitory signaling, combining checkpoint blockade with a co-stimulatory intervention may be rational. By contrast, tumors with defective priming, poor T-cell infiltration, or marked metabolic suppression require concurrent strategies that address these barriers, because additional checkpoint blockade or systemic co-stimulatory agonism alone is unlikely to restore durable CTL activity [27, 109].

4-1BB agonism represents one approach to reinforce CTL survival and effector function when checkpoint inhibition alone is insufficient. In immunocompetent B-cell lymphoma models, PD-L1 blockade combined with a 4-1BB agonist enhanced intratumoral CD8+ T-cell activity and prolonged survival relative to either monotherapy [125]. This evidence is preclinical. In a small phase Ib study of utomilumab plus pembrolizumab in advanced solid tumors, the combination showed acceptable tolerability and preliminary antitumor activity, but its clinical benefit has not been established in randomized trials [126]. These limitations have motivated the development of tumor-localized or conditionally active 4-1BB agonists intended to improve the therapeutic window [127].

Bispecific and trispecific antibody formats should be classified according to the signals they deliver. Conventional tumor antigen×CD3 engagers redirect and activate T cells through CD3, but they do not reproduce the B7–CD28 signal required for conventional co-stimulation. Tumor-targeted CD28 bispecific antibodies can provide a conditional co-stimulatory input when combined with tumor-directed CD3 engagement, although the supporting evidence remains preclinical [15]. CD38×CD3×CD28 trispecific engagers have likewise enhanced T-cell activation and cytotoxicity in preclinical multiple myeloma systems [128]. These platforms should therefore be regarded as preclinical or early translational co-stimulatory strategies rather than clinically established alternatives to approved checkpoint therapies.

Cytokine-based strategies can complement, but do not substitute for, deficient co-stimulation. IL-12 can support CD8+ T-cell activation and reshape suppressive tumor microenvironments, but systemic exposure constrains its therapeutic application. Preclinical approaches that localize or conditionally deliver IL-12, including PD-1-targeted low-affinity IL-12, IL-12 prodrugs, and cell surface–tethered IL-12, have enhanced intratumoral immune activity while aiming to limit systemic exposure [77–79]. In addition, intratumoral plasmid IL-12 combined with pembrolizumab has shown phase II clinical activity in immunologically quiescent metastatic melanoma [129]. This result provides a tumor-context-specific clinical signal, but the non-randomized design does not establish broad clinical validation of IL-12-based combination therapy across tumor types.

Across all combination classes, dose, schedule, route of delivery, tumor-targeting specificity, and evidence level should inform therapeutic prioritization and clinical evaluation. Checkpoint blockade, co-stimulatory agonists, targeted multispecific formats, and cytokine support are most likely to be useful when each component corrects a defined barrier to tumor-reactive CD8+ T-cell priming, local reactivation, infiltration, or persistence.

### Gene- and nucleic acid–based restoration of co-stimulatory signals

Gene- and nucleic acid–based approaches can restore or bypass deficient co-stimulatory signaling by engineering therapeutic T cells or by delivering co-stimulatory ligands locally within tumors. These approaches are mechanistically distinct and should not be treated as a single evidence category.

CAR T-cell design provides the clearest example of co-stimulatory engineering. CARs incorporating CD28 or 4-1BB endodomains exhibit distinct activation and metabolic programs: CD28-based CARs favor early glycolytic effector responses, whereas 4-1BB-based CARs favor mitochondrial metabolism and memory-associated differentiation [42]. Rather than simply increasing signal strength, the choice of co-stimulatory domain calibrates the balance between rapid effector activity and persistence. Genome editing represents a related but distinct strategy for improving engineered T-cell fitness. Targeted insertion of a CAR into the TRAC locus reduced tonic signaling and enhanced tumor rejection in preclinical models, illustrating how receptor-expression control can improve engineered T-cell function without directly replacing co-stimulatory domain selection [130].

Local nucleic acid delivery represents a complementary strategy for increasing co-stimulatory ligand availability within tumors. In mouse lymphoma and colon carcinoma models, intratumoral delivery of mRNA encoding OX40L, CD80, and CD86 induced local T-cell activation and systemic antitumor responses [131]. This approach remains preclinical and should be viewed as a tumor-localized strategy to improve the local activation environment rather than as an established clinical method of restoring APC function.

Chimeric switch receptors provide a different form of co-stimulatory engineering. In preclinical melanoma systems, a Siglec-7 extracellular domain fused to a 4-1BB intracellular domain was co-expressed with a MART-1-specific TCR, converting recognition of tumor-associated sialylated ligands into a co-stimulatory signal [132]. This design enhanced cytokine secretion and tumor control while retaining dependence on concurrent antigen-specific TCR activation [132]. It therefore supplements, rather than replaces, Signal 1.

**Fig. 4:**
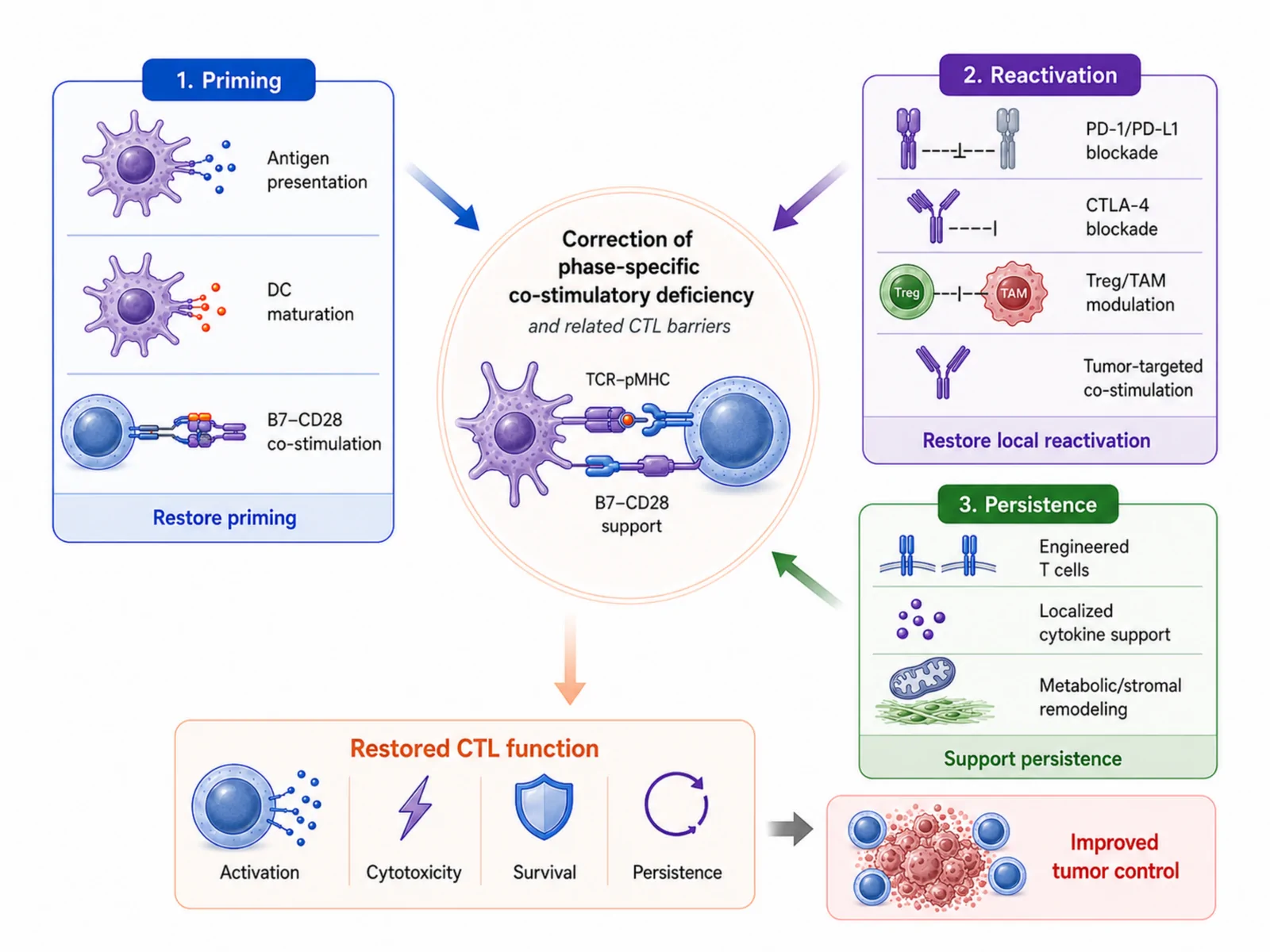
Mechanism-matched therapeutic strategies for phase-specific co-stimulatory deficiency. Therapeutic interventions should be matched to the phase at which CTL responses are predominantly impaired. Priming-directed strategies aim to improve antigen presentation, dendritic-cell maturation, and B7–CD28 co-stimulation. Reactivation-directed strategies relieve PD-1/PD-L1 and CTLA-4 inhibition, modulate Treg/TAM-mediated suppression, and conditionally enhance local co-stimulation. Persistence-directed strategies include engineered T-cell approaches, localized cytokine support, and metabolic or stromal remodeling. Together, these approaches aim to restore CTL activation, cytotoxicity, survival, and persistence, thereby improving tumor control. Evidence maturity and translational constraints are summarized in Table 2

Gene- and nucleic acid–based approaches should be matched to the dominant defect within each tumor context. CAR co-stimulatory design and genome editing are most relevant when signaling calibration, resistance to tonic activation, or therapeutic T-cell persistence is limiting, whereas local delivery of co-stimulatory ligands may be more relevant when the tumor microenvironment lacks adequate activating input. Their translational use should also reflect the phase at which CTL function is impaired: inadequate priming, restricted local reactivation, or poor long-term persistence. Across these approaches, tumor selectivity, control of signal intensity, and the distinction between clinical, early clinical, and preclinical evidence remain critical considerations. The phase-matched therapeutic logic linking impaired priming, intratumoral reactivation, and CTL persistence is illustrated in Fig. 4. Therapeutic strategies to restore, bypass, or engineer co-stimulatory signaling are summarized in Table 2 according to their biological rationale, evidence maturity, and translational constraints.

**Table 2:** Therapeutic approaches to restore, bypass, or engineer co-stimulatory signaling in cancer immunotherapy

Therapeutic modality and representative approach	Intended co-stimulatory defect or biological rationale	Most suitable tumor or immune context	Evidence maturity	Major safety, feasibility, or implementation constraints
PD-1/PD-L1 blockade	Releases PD-1-mediated suppression of residual CD28-dependent CTL reactivation [26, 27]	Checkpoint-dominant tumors with pre-existing tumor-reactive CTLs, preserved antigen presentation, and residual APC/B7 support	Established clinical efficacy in selected tumor types [104,106–108].	Limited benefit in immune-cold, APC-defective, or profoundly T-cell-depleted tumors; PD-L1 alone is insufficient for patient selection
CTLA-4 blockade, alone or combined with PD-1/PD-L1 blockade	Can preserve B7 availability by counteracting CTLA-4-mediated competition and removal of CD80/CD86; may attenuate Treg-mediated co-stimulatory restriction.	Treg-rich or B7-restricted tumor contexts with retained APC competence and tumor-reactive T cells.	Established clinical efficacy in selected indications; combination benefit demonstrated in selected melanoma and NSCLC settings [64,101,102].	Increased immune-related adverse events; efficacy depends on recoverable APC and T-cell compartments rather than checkpoint expression alone
APC- and priming-restoration strategies, including DC vaccination, DC maturation or recruitment approaches, innate immune activation, and restoration of tumor antigen presentation	Restores reversible defects in antigen presentation, dendritic-cell recruitment or maturation, and B7–CD28 priming conditions required for productive CTL activation.	Immune-cold or priming-defective tumors with inadequate antigenicity, cDC1 recruitment, dendritic-cell maturation, or reversible impairment of MHC class I antigen presentation.	Predominantly preclinical/translational or early clinical; clinical benefit remains platform- and disease-specific [55,56,121].	Heterogeneous platforms, variable antigen selection, inconsistent DC quality, limited trafficking, and uncertain optimal combination partners
4-1BB agonism and conditionally active tumor-localized 4-1BB platforms	Reinforces activated CTL survival, cytotoxicity, metabolic fitness, and persistence after antigen encounter.	Tumors with residual tumor-reactive T cells but insufficient effector maintenance or persistence	Early clinical evidence exists for systemic 4-1BB agonism; conditionally active tumor-localized platforms remain mainly preclinical or early clinical, with no broadly established efficacy [125–127].	Systemic agonism may have a narrow therapeutic window, including hepatotoxicity; activity depends on adequate target localization, conditional receptor engagement, and a pre-existing T-cell response.
OX40 agonistic strategies	Provides context-dependent reinforcement of T-cell expansion, survival, and memory-associated persistence after activation.	Tumor contexts with retained antigen-reactive T cells but incomplete activation or persistence; most appropriately considered in rational combination strategies.	Preclinical and early translational; no broadly established clinical efficacy across solid tumors [13,38].	Monotherapy activity is often limited; receptor expression, treatment timing, Fc biology, and combination-partner selection are critical.
Tumor-targeted CD28 co-stimulators and dual-signal CD3/CD28-engaging formats	Couples CD28 activation to tumor antigen recognition, PD-L1 expression, or simultaneous TCR/CD3 engagement to avoid nonspecific systemic activation	Tumors with accessible target antigens, CD28-expressing T cells, and insufficient local co-stimulatory input.	Preclinical to early translational; no established clinical efficacy [15].	Severe cytokine-release risk with uncontrolled systemic CD28 activation; requires stringent conditional activation, antigen specificity, and dose optimization
T-cell-redirecting antibody combinations or multispecific formats incorporating a co-stimulatory module	Combines tumor-cell recognition and CD3-mediated activation with conditional CD28 or 4-1BB support, thereby reducing dependence on endogenous APC-derived co-stimulation.	Especially relevant in antigen-defined hematologic malignancies; investigational in solid tumors with accessible tumor-associated antigens	Formats incorporating an added co-stimulatory module remain preclinical or early translational [15,128].	Cytokine release syndrome, neurotoxicity, on-target/off-tumor effects, antigen heterogeneity, and limited penetration into solid tumors
Engineered T-cell strategies, including CD28- or 4-1BB-based CAR-T cells, TCR-T cells, switch receptors, and gene-edited T cells	Incorporates or rewires co-stimulatory signals to reduce dependence on endogenous APC-derived support and improve activation, persistence, and resistance to local suppression.	Hematologic malignancies with defined target antigens; selected solid-tumor settings requiring enhanced persistence, trafficking, or resistance to suppression	CAR-T clinically established mainly in hematologic malignancies; TCR-T and next-generation engineered platforms are early clinical or investigational in most solid tumors [41,42,130–132].	Antigen heterogeneity, impaired trafficking, poor persistence, suppressive stromal/myeloid microenvironments, cytokine toxicity, neurotoxicity, and off-tumor recognition
TME-remodeling and metabolic-restoration strategies, including TAM/CAF reprogramming, vascular normalization, hypoxia/lactate targeting, and metabolic-stress modulation	Improves APC recruitment, T-cell access, local reactivation, and metabolic support rather than directly replacing B7–CD28 co-stimulation.	Stromal-, metabolic-, or myeloid-constrained tumors with incomplete local CTL reactivation and poor persistence	Mixed evidence; disease-specific combinations range from clinical use to preclinical investigation [110,111,119,120].	Mechanistic heterogeneity, lack of validated biomarkers, compensatory suppression, and difficulty demonstrating that clinical benefit derives specifically from restored co-stimulatory competence

Abbreviations: APC, antigen-presenting cell; CAF, cancer-associated fibroblast; CTL, cytotoxic T lymphocyte; DC, dendritic cell; TAM, tumor-associated macrophage; TCR-T, T-cell receptor-engineered T-cell; TME, tumor microenvironment

Evidence maturity is summarized descriptively and may combine established clinical, clinical/early clinical, and preclinical/translational evidence where applicable. This classification refers to the maturity of each therapeutic modality or platform and does not indicate validation of a specific co-stimulatory biomarker for routine clinical patient selection

## Future perspectives

Co-stimulatory failure should not be regarded as a uniform defect across tumors or across stages of the CTL response. At the priming stage, impaired dendritic-cell maturation, restricted B7 availability, and deficient antigen presentation can prevent the generation of an effective tumor-reactive CTL pool. These mechanisms are related but not equivalent: antigen-presentation defects act upstream of co-stimulation, whereas inadequate B7–CD28 signaling directly limits the second signal required for productive activation. Within established tumors, CTLA-4-mediated restriction of B7 availability, PD-1-mediated inhibition of residual CD28-dependent signaling, and suppressive myeloid or regulatory-cell programs may impair local CTL reactivation. During prolonged tumor exposure, metabolic stress, stromal exclusion, and inadequate survival support can further compromise CTL persistence. Longitudinal and spatially resolved studies will be needed to determine when and where these distinct defects emerge during tumor progression and treatment. Because the cancer-immunity cycle is iterative, with tumor-cell killing capable of initiating subsequent rounds of antigen presentation and T-cell stimulation [133], an additional priority is to determine whether local co-stimulatory support is exclusively APC-derived or can, in defined settings, be supplemented by tumor-cell-associated ligands with co-stimulatory potential. Such studies should map the cellular sources and spatial distribution of these candidate ligands, determine whether cognate receptor–ligand interactions are accessible to tumor-reactive CTLs, and test the consequences of ligand perturbation for local CTL activation and response to checkpoint blockade.

At present, however, the available literature does not provide a sufficiently harmonized cross-tumor evidence base from which defensible numerical prevalence estimates for priming-, intratumoral reactivation-, and persistence-phase co-stimulatory defects can be derived. Published studies frequently assess non-equivalent proxies—including antigen-presentation competence, dendritic-cell maturation, CD80/CD86 availability, checkpoint signaling, or dysfunctional T-cell states—across different tumor types, tissue compartments, treatment settings, and sampling time points. These variables cannot yet be combined into a unified quantitative estimate of the relative prevalence of co-stimulatory defects across CTL-response phases.

A major priority is to distinguish mechanistically informative tumor-immune states from clinically validated biomarkers for patient selection. The categories proposed in Table 1 should be viewed as analytical and non-mutually exclusive contexts rather than fixed histological classifications or validated treatment-selection groups. Prospective studies should integrate tumor antigen-presentation status, dendritic-cell differentiation and CD80/CD86 availability, the spatial organization of APC–CTL interactions, the expression and spatial accessibility of candidate tumor-cell-associated ligand–receptor pairs with co-stimulatory potential, intratumoral Treg and myeloid-cell states, CTL differentiation profiles, checkpoint-ligand distribution, stromal architecture, and metabolic stress signatures. Such profiling may help determine whether a tumor is predominantly checkpoint-dominant, priming-defective, B7-restricted, immune-excluded, metabolically constrained, or persistence-limited. At present, neither an individual component nor the composite framework should be interpreted as a clinically validated biomarker algorithm.

Therapeutic development should be guided by the dominant barrier to CTL function rather than by empirical escalation of immunotherapy. Checkpoint-dominant tumors with pre-existing tumor-reactive T cells and residual APC support may be more suitable for inhibitory-checkpoint blockade. By contrast, Treg-rich or B7-restricted contexts may require preservation of APC-derived co-stimulation or attenuation of Treg-mediated suppression; immune-cold or priming-defective tumors may first require restoration of antigenicity, dendritic-cell recruitment or maturation, and productive T-cell trafficking. In persistence-limited settings, engineered T cells, conditional co-stimulatory platforms, or localized cytokine support may be more relevant than additional checkpoint inhibition alone. The clinical maturity of these approaches remains heterogeneous and ranges from established checkpoint therapies to early clinical, translational, and preclinical strategies.

Future trials should test this framework prospectively using baseline and on-treatment samples. Relevant measurements include antigen-presentation competence, APC co-stimulatory capacity, spatial proximity between APCs and tumor-reactive CTLs, expression, cognate receptor engagement, and functional accessibility of candidate tumor-cell-associated ligands with co-stimulatory potential, CTL proliferation and persistence, inhibitory-receptor signaling, stromal architecture, and metabolic constraints. The key question is not simply whether a combination enhances immune activity, but whether it corrects the dominant defect in productive priming, locally accessible co-stimulatory support for CTL reactivation, or long-term persistence. Such mechanism-oriented designs may improve combination selection while avoiding added toxicity from interventions that do not address the limiting step in a given tumor.

## Conclusion

Effective CTL-mediated tumor control requires coordinated antigen recognition, co-stimulatory input, and sustained support within the tumor microenvironment. Deficient B7–CD28 co-stimulation does not produce a single uniform form of CTL dysfunction. At antigen encounter, it can favor anergy-like hyporesponsiveness and inadequate clonal expansion; insufficient pro-survival support can increase vulnerability to apoptotic attrition; and persistent antigen exposure together with sustained inhibitory signaling can promote exhaustion-associated dysfunction. These states overlap but are not interchangeable, and their distinction is essential for selecting mechanism-matched therapeutic interventions.

Tumors can impair productive CTL responses through a combination of antigen-presentation failure, defective APC differentiation, reduced B7 availability, CTLA-4-mediated competition for and removal of CD80/CD86, PD-1-mediated inhibition of residual CD28-dependent signaling, and suppressive Treg, myeloid, stromal, and metabolic programs. Antigen-presentation failure is an upstream and distinct barrier to CTL activation, whereas reduced B7 availability and APC dysfunction directly constrain co-stimulatory support. This framework helps explain why checkpoint blockade alone may be insufficient when tumor-reactive CTLs are absent, poorly primed, excluded from the tumor bed, metabolically constrained, or unable to persist after activation. Conversely, checkpoint blockade is more likely to be effective when antigen-specific T cells, functional antigen presentation, and residual co-stimulatory capacity are retained.

The tumor-context-specific framework presented in Table 1 and the therapeutic evidence hierarchy summarized in Table 2 provide a mechanism-based basis for therapeutic prioritization and hypothesis generation. They are not yet validated tools for clinical treatment selection. Rather than applying combination immunotherapy empirically, future strategies should be matched to the dominant barrier to CTL function, including deficient priming, B7 restriction, checkpoint dominance, immune exclusion, metabolic stress, or impaired persistence. Durable anti-tumor immunity will likely require coordinated restoration of the activation, survival, and locally accessible co-stimulatory support needed for CTLs to remain effective within suppressive tumor microenvironments, together with a clearer definition of the cellular sources capable of sustaining such support.

## Data Availability

Data sharing is not applicable to this article, as no datasets were generated or analyzed during the current study.
